# Supplementary material for: Serum N-Glycosylation RPLC-FD-MS Assay to Assess Colorectal Cancer Surgical Interventions
Source: Biomolecules. 2023 May 27;13(6):896. doi: 10.3390/biom13060896 (PMC10295937; doi:10.3390/biom13060896)
Supplement: Supplementary file 1 [file biomolecules-13-00896-s001.zip › 02_CRC_SI_mm1_09May23.pdf]

Supplementary Materials

Serum *N*-glycosylation RPLC-FD-MS Assay to Assess Colorectal Cancer Surgical Interventions

Alan B. Moran[1,2], Georgia Elgood-Hunt[2], Yuri E.M. van der Burgt[1], Manfred Wuhrer[1], Wilma E. Mesker[3], Rob A.E.M. Tollenaar[3], Daniel I.R. Spencer[2], Guinevere S.M. Lageveen-Kammeijer[1,4] *

1 Leiden University Medical Center, Center for Proteomics and Metabolomics, 2300 RC Leiden, The Netherlands

2 Ludger Ltd., Culham Science Centre, OX14 3EB Abingdon, United Kingdom

3 Leiden University Medical Center, Department of Surgery, 2300 RC Leiden, The Netherlands

4 University of Groningen, Department of Analytical Biochemistry, Groningen Research Institute of Pharmacy, Groningen, The Netherlands

* **Correspondence**: Guinevere S.M. Lageveen-Kammeijer, Leiden University Medical Center, Center for Proteomics and Metabolomics, P.O. Box 9600, 2300 RC Leiden, The Netherlands and University of Groningen, Department of Analytical Biochemistry, Groningen Research Institute of Pharmacy, 9713 AV Groningen, The Netherlands; g.s.m.kammeijer@rug.nl

Table of Contents

Supplementary Materials S-1

Supplementary Materials – Figures S-2

Figure S1. Representative *N*-glycan profile measured by RPLC-FD-MS. S-2

Figure S2. Extracted ion chromatograms of H4N4F1 from IgG S-3

Figure S3. Differences in histological type between pre- and post-operative samples measured by MALDI-MS S-4

Figure S4. Significant *N*-glycans for histological type in MALDI-MS discovery and validation study S-5

Figure S5. Linear regression comparison S-6

References S-7

Supplementary Materials – Figures

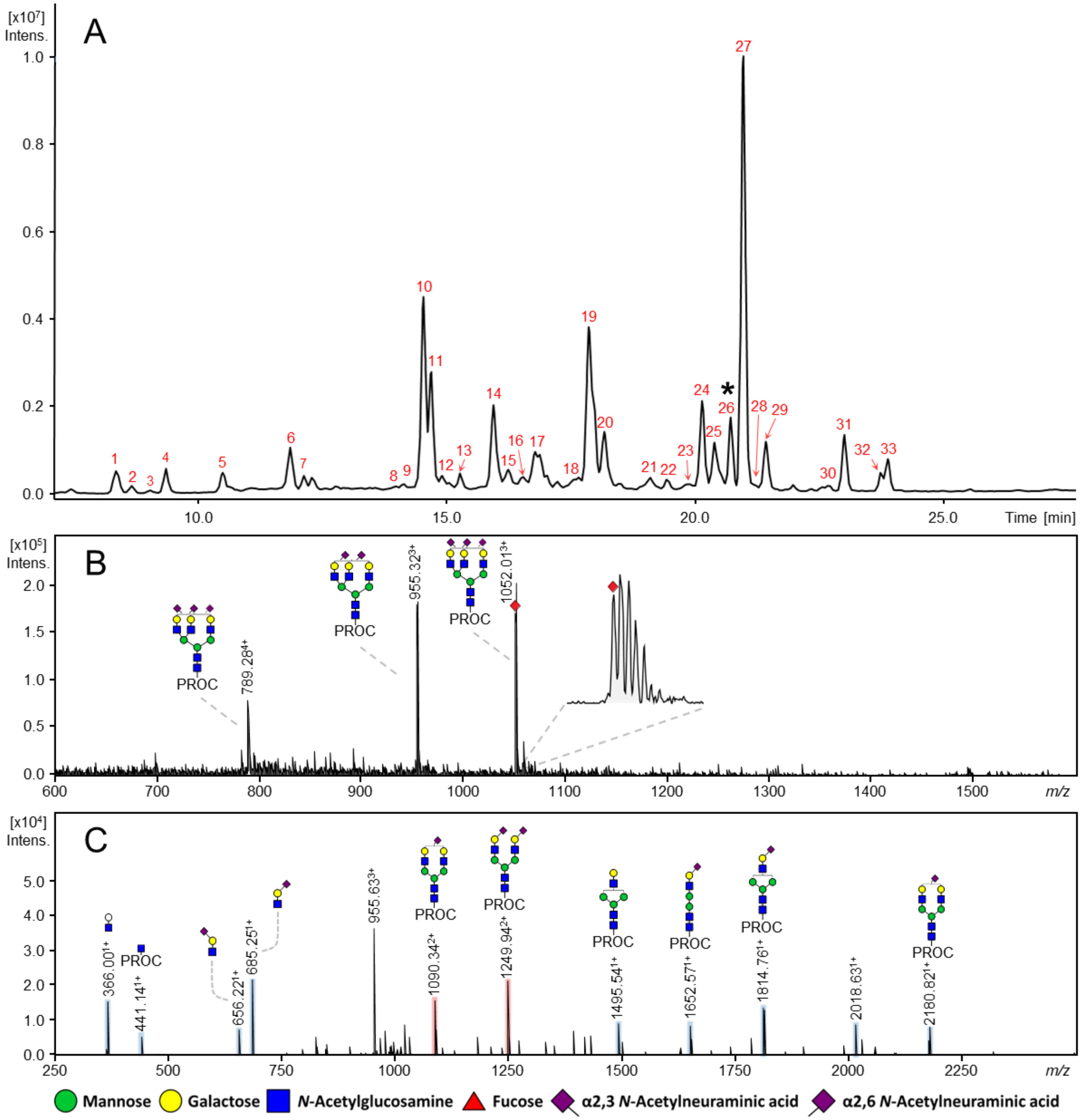

Figure S1. Representative *N*-glycan profile measured by RPLC-FD-MS. (A) Fluorescent chromatogram with peaks numbered from 1 – 33. Asterisk denotes that peak 26 is shown is panel B. **(B)** MS spectrum of peak 26 whereby the assignments of H6N5S$_{2,3}$1S$_{2,6}$2 (isomer 2) (*m/z* 789.28^{4+}, 1052.01^{3+}) as well as H6N5S$_{2,6}$2 (isomer 1) (*m/z* 955.32^{3+}) are shown. An inset with a close-up of the isotopic profile *m/z* 1052.01^{3+} is provided. The red diamond indicates the precursor that was selected for MS/MS. **(C)** MS/MS spectrum of *m/z* 1052.01^{3+} with annotated fragment ions [M+H]$^{1+}$ (blue) and [M+2H]$^{2+}$ (red).

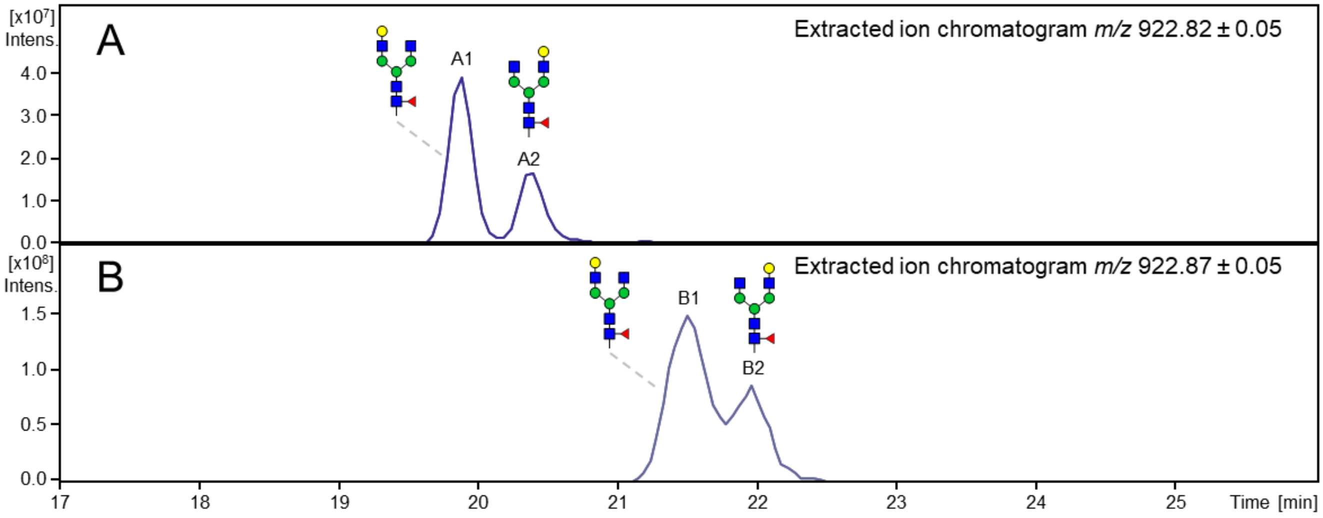

Figure S2. Extracted ion chromatograms of H4N4F1 from IgG. The extracted *m/z* of $[M+2H]^{2+}$ shown. **(A)** HILIC separation of these glycoforms following procainamide labeling. The assignment of the isomers in peaks A1 and peak A2 has been reported in the literature [1]. The gradient consisted of: 0 to 53.5 min, 76 to 55% B (0.4 mL/min); 53.5 to 54.5 min, 55 to 0% B (0.4 to 0.2 mL/min); 54.5 to 57.5 min, 0% B (0.2 mL/min); 57.5 to 59.5 min, 0 to 76% B (0.2 mL/min); 59.5 to 70 min, 76% B (0.2 to 0.4 mL/min). **(B)** RPLC separation following procainamide labeling and sialic acid ethyl esterification and amidation. Peaks B1 and B2 are tentatively assigned based on the precursor *m/z* and relative peak ratio, which is similar to (A). The following gradient was used: 0 to 53.5 min, 40 to 80% B; 53.5 to 57.5 min, 80% B; 57.5 to 59.5 min, 80 to 40% B; 59.5 to 70.1 min, 40% B; 0.2 mL/min.

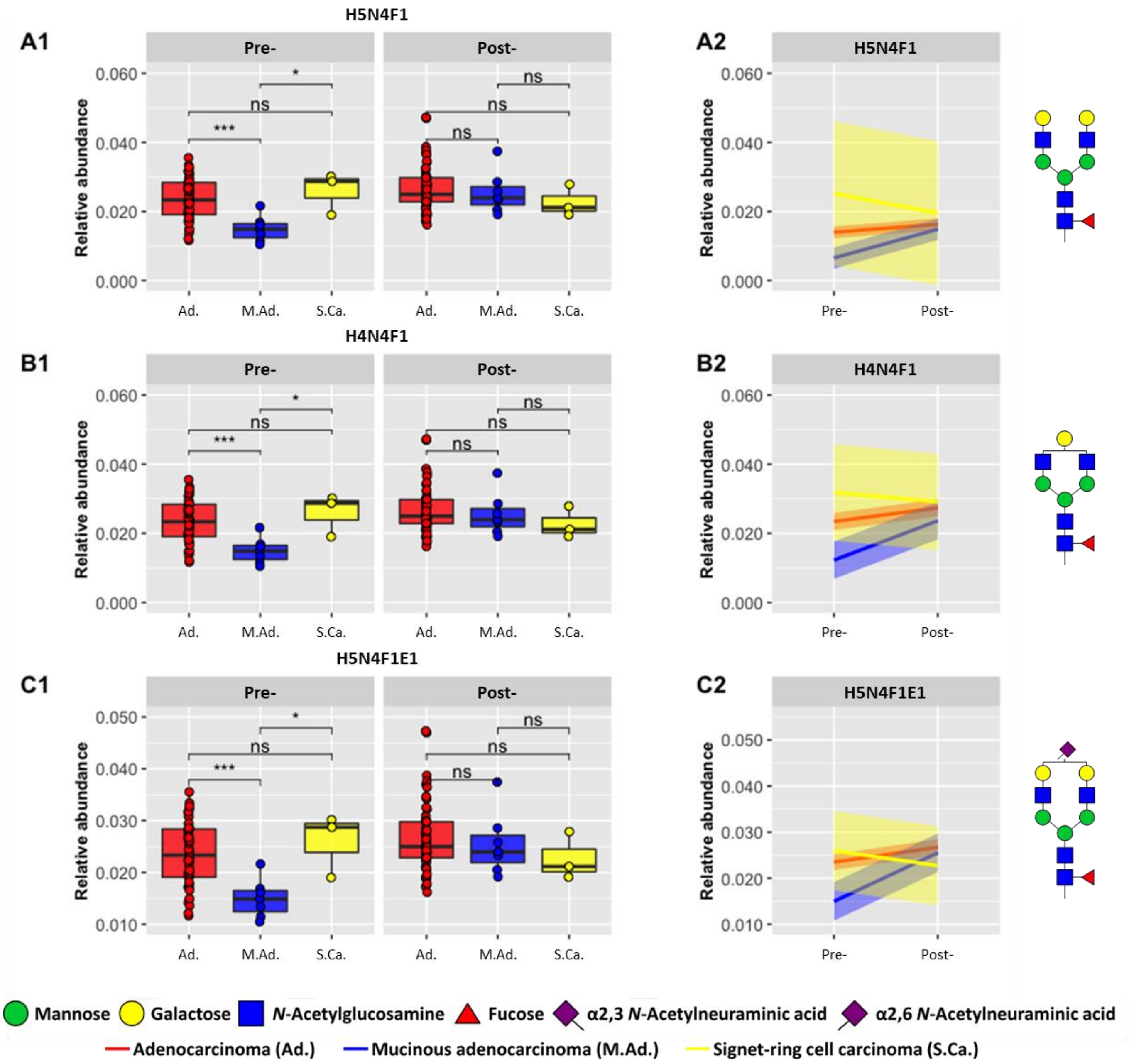

Figure S3. Differences in histological type between pre- and post-operative samples measured by MALDI-MS. The eight significant *N*-glycans found by RPLC-FD-MS were assessed in the pre- and post-operative MALDI-MS data set. [2] Three *N*-glycans differentiated significantly based upon histological type, adenocarcinoma (n = 57), mucinous adenocarcinoma (n = 7) and signet-ring cell carcinoma (n = 3), as denoted by each row: Row A (H5N4F1), row B (H4N4F1) and row C (H5N4F1$S_{2,6}$1). **(1)** Boxplots of relative abundances observed in pre- and post-operative samples. **(2)** Trend observed following surgery, from pre- to post-operative samples from the same patients. The 95% CI is shown as colored bands around each line. Asterisks denote significance with p-value < 0.05 (*), 0.01 (**), 0.001 (***), 0.0001 (****). Abbreviations: Not significant (ns), hexose (H), *N*-acetylhexosamine (N), fucose (F), amidated α2,3-linked *N*-acetylneuraminic acid (Am) and ethyl esterified α2,6-linked *N*-acetylneuraminic acid (E).

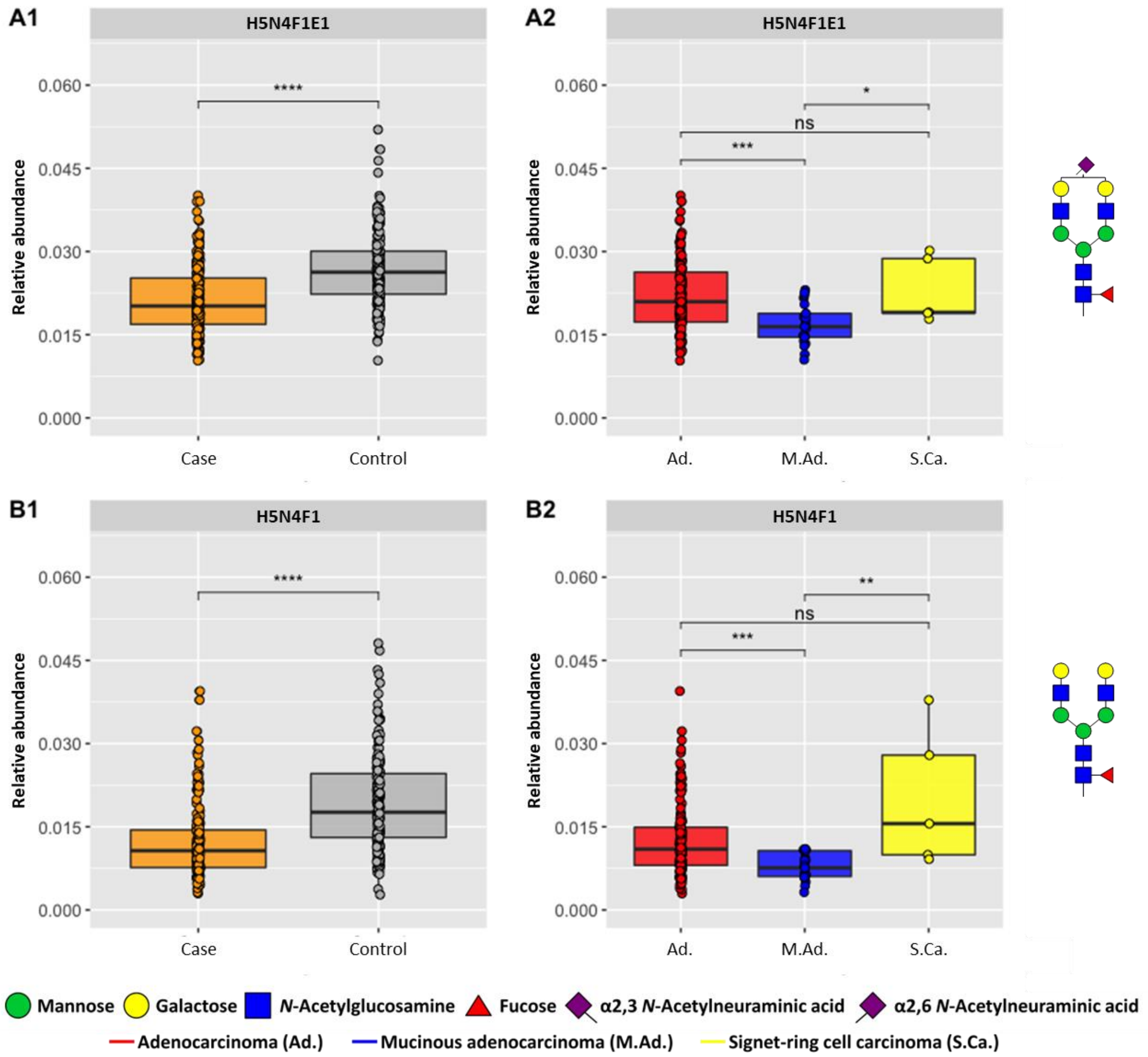

Figure S4. Significant *N*-glycans for histological type in MALDI-MS discovery and validation study. The eight significant *N*-glycans found by RPLC-FD-MS were assessed between cases (n = 185) and controls (n = 185) in the discovery and validation MALDI-MS data set. [2] Two *N*-glycans differentiated significantly based upon histological type, adenocarcinoma (n = 154), mucinous adenocarcinoma (n = 21) and signet-ring cell carcinoma (n = 7), as denoted by rows A (H5N4F1$S_{2,6}$1) and B (H5N4F1). **(1)** Boxplots of relative abundances observed in cases *versus* healthy controls (n = 185). **(2)** Boxplots of relative abundances (cases) observed across the three histological types. Metadata was not collected in five patients, therefore the number of plotted cases is n = 180. Asterisks denote significance with p-value < 0.05 (*), 0.01 (**), 0.001 (***), 0.0001 (****). Abbreviations: Not significant (ns), hexose (H), *N*-acetylhexosamine (N), fucose (F), amidated α2,3-linked *N*-acetylneuraminic acid (Am) and ethyl esterified α2,6-linked *N*-acetylneuraminic acid (E).

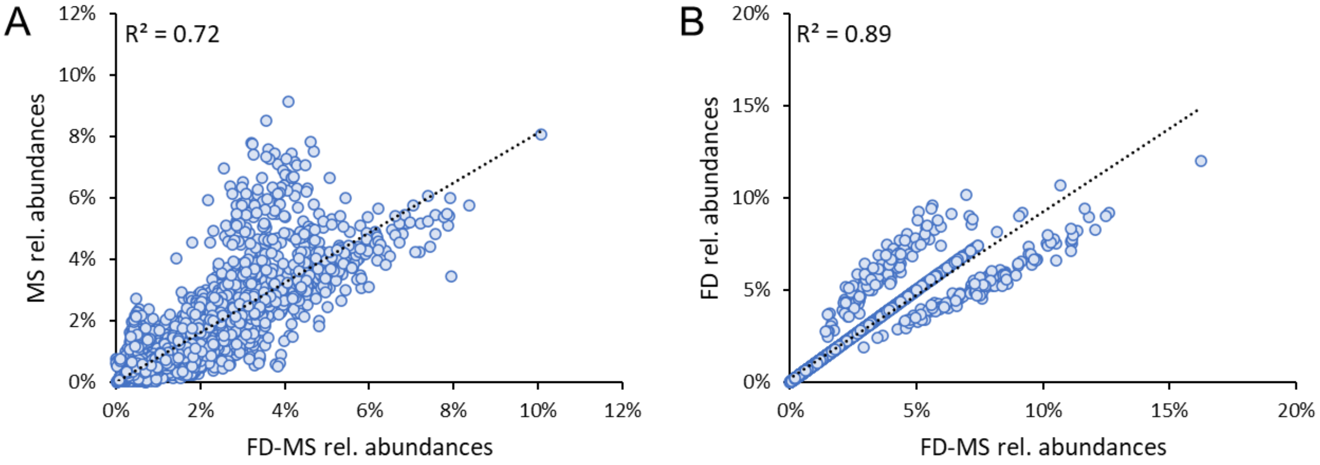

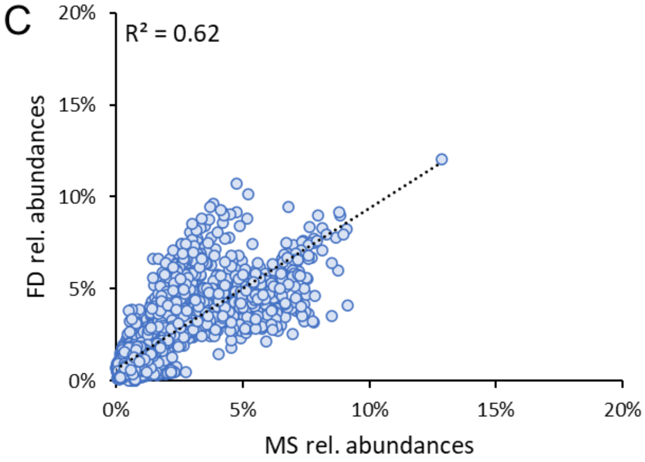

D

Comparison	Analytes[1,2]	Measurements	R^2 A	R^2 B
FD-MS vs. MS	48	128	0.94	0.72
FD-MS vs. FD	32	128	0.98	0.89
MS vs. FD	32	128	0.91	0.62

[1] 48 *N*-glycans determined by MS were compared
[2] 32 analytes were compared whereby MS signals for *N*-glycans eluting under the same FD peak were summed to compare with FD. FD peaks 2 and 3 were summed
A All analytes included
B Excluding the two most abundant analytes

Figure S5. Linear regression comparison. (A) Comparison of FD-MS and MS relative abundances. **(B)** Comparison of FD-MS and FD relative abundances. A discrepancy may have been introduced due to batch correction which was performed after FD-MS calculation **(C)** Comparison of MS and FD relative abundances. **(D)** Summary table of all comparisons. *N*-glycans signals eluting under the same fluorescent peak were summed in order to perform the comparison with FD quantification. Similarly, FD peaks 2 and 3 were summed as H7N2 isomers were quantified as one signal by MS quantification approaches. Correlation between quantification approaches is given via the R^2 values, colored from 0 (red) to 0.5 (yellow) to 1 (green).

References

1. Keser, T.; Pavić, T.; Lauc, G.; Gornik, O. Comparison of 2-Aminobenzamide, Procainamide and RapiFluor-MS as Derivatizing Agents for High-Throughput HILIC-UPLC-FLR-MS N-Glycan Analysis. *Front Chem* **2018**, *6*, doi:10.3389/fchem.2018.00324.
2. de Vroome, S.W.; Holst, S.; Girondo, M.R.; van der Burgt, Y.E.M.; Mesker, W.E.; Tollenaar, R.A.E.M.; Wuhrer, M. Serum N-Glycome Alterations in Colorectal Cancer Associate with Survival. *Oncotarget* **2018**, *9*, 30610–30623, doi:10.18632/oncotarget.25753.
